# Levels of salivary immunoglobulins and periodontal evaluation in smoking patients

**DOI:** 10.1186/1471-2172-15-5

**Published:** 2014-02-06

**Authors:** Maria Rita Giuca, Marco Pasini, Simona Tecco, Giacomo Giuca, Giuseppe Marzo

**Affiliations:** 1 Dipartimento di Patologia Chirurgica, Medica, Molecolare e dell’Area Critica, University of Pisa, Via Savi 10, Pisa IT 56126, Italy; 2 University of L’Aquila, School of Orthodontics, Pescara, Via Le Mainarde 26, Pescara IT 65124, Italy; 3 Department MeSVA, University of L’Aquila, Edificio Delta 6, Coppito, L’Aquila IT 67010, Italy

## Abstract

**Objective:**

The aim of this study was to assess the level of salivary immunoglobulins and periodontal status in smokers and non-smokers.

**Materials and methods:**

Unstimulated saliva of 30 subjects (mean age 24.2 ± 3.5 years) who were smokers (test group) and of 30 subjects (mean age 25.3 ± 3.8 years) who were non-smokers (control group) was collected and centrifugated; IgA, IgG, and IgM were measured with the colorimetric immunoenzymatic method. Moreover, the following periodontal clinical parameters were recorded for each subject: plaque index (PI), gingival index (GI), probing depth (PD), and clinical attachment level (CAL).

**Results:**

A significantly (*p*< 0.05) lower Ig level was observed in smoking patients (IgA: 20.0 ± 1.2 mg/dl; IgM: 19.5 ± 1.6 mg/dl; IgG: 8.1 ± 1.4 mg/dl) compared to levels in the non-smoking control group (IgA: 234.1 ± 65.2 mg/dl; IgM: 121.0 ± 31.7 mg/dl; IgG: 1049.4 ± 102 mg/dl). In the test group, PI (2.2 ± 0.3), GI (2.4 ±0.5), PD (49.3 ± 9.2%), and CAL (49.3 ± 4.6%) were higher (*p*< 0.05) than those observed in the control group (PI: 0.8 ± 0.4; GI: 0.7 ± 0.3; PD: 10.6 ± 2.4%; CAL: 3.1 ± 0.8%).

**Conclusion:**

Smoking subjects showed lower levels of salivary IgA, IgG, and IgM and a worse periodontal condition than non-smoking subjects. On the base of our study, as smoking subjects also had lower levels of IgA, IgG, and IgM in their saliva than non-smoking subjects, despite the fact that there is little evidence that the salivary Igs have a protective action against periodontitis and that the whole saliva does not result in whole from the salivary glands, it can be concluded that the deteriorated periodontal health conditions of these patients can be attributed in part to a lowering of the host’s defense due to a decrease in the quantity of Igs in salivary fluid.

## Background

Immunoglobulins (Igs) are protein molecules produced by special cells in organisms’ immune systems in response to the presence of the penetration of external agents, such as viruses, bacteria, protozoans, fungi, tumor cells, or tissues that are recognized as foreign because of the presence of cell surface antigens [1]. The function of Igs is to bind antigen molecules with specificity and, consequently, target bound molecules, such as toxins and constituents of micro-organisms and parasites, for inactivation and/or elimination from the organism [2].

Five Ig classes have been distinguished: IgMs, which are the first type of antibody that neonates can produce in case of an infection; IgGs, which appear when the organism is exposed for the second time to a particular antigen and provide protection against toxins and viruses; IgEs, which are produced after an allergic reaction and function to protect the organism against parasite infections; IgAs, which protect the organism against local infections; and IgDs, which act as receptors for B lymphocyte antigens, although their role has not been completely clarified yet [3].

Igs are also present in saliva, where they act to provide protection of the oral cavity. In particular, IgAs, which are produced by the plasma cells of the salivary glands, are the most represented Ig type in salivary fluid and, together with the action carried out by the subgingival microflora, exert a protective action against the oral bacteria [1,2].

It has been observed that cigarette smoking can alter the salivary system by increasing salivary fluid, reducing certain salivary enzymes (i.e., amylase, lactic dehydrogenase, and acid phosphatase), and altering anti-oxidizing enzymes (i.e. glutathione peroxidase) and immune system function [4,5]. Hence, a reduction of the Igs contained in saliva can represent an increased risk factor for the host mucosa with respect to pathogenic microorganisms, including periodontal pathogens [6]. Although bacteria are the main etiological factor in the appearance of periodontal diseases, the individual’s response is a crucial factor in his or her susceptibility to disease [7]. Smoking can worsen one’s periodontal status by altering the defenses through two different mechanisms: damaging the host’s normal response in the neutralization of infections and producing alterations that cause the destruction of surrounding healthy periodontal tissues [8].

Salivary immunoglobulin levels in smoking patients with periodontal disease have not yet been studied comprehensively in literature.

The purpose of this study was to evaluate whether a decrease in the levels of immunoglobulins (IgA, IgG, IgM) in smokers is related to a worsening of the parameters of periodontal health.

## Methods

### Selection criteria of test patients and controls

A total of 60 patients were included in this pilot study. A written consent was obtained from all patients for their participation in the study. All of the procedures were carried out in compliance with the Declaration of Helsinki. This study was approved by the ethical committee.

All of the patients racially identified themselves as Caucasian (white) and had their complete set of permanent teeth. According to our anamnesis assessments, all showed similar habits with regard to nutrition and oral hygiene (use of manual toothbrush and toothpaste only, twice a day, and no use of other hygiene tools such as dental floss, an interdental brush, or mouthwash). The exclusion criteria were as follows: systemic diseases; chronic use of drugs that could alter periodontal conditions; having received professional hygiene or periodontal therapies in the 6 months preceding the screening; orthodontic care in progress; or dental fractures with a score higher than 1, according to the classification of Ellis [9].

The test group was composed of 30 individuals (15 males and 15 females; mean age 24.2 ± 3.5 years) who were smokers (≥20 cigarettes/day for the last 4 years or longer). The young age of the participants is justified as we wanted to investigate these patients for the presence of early-onset periodontal disease.

The control group was composed of 30 individuals (15 males and 15 females; mean age 25.3 ± 3.8 years) who were non-smokers (they declared they had never smoked in their entire lives).

### Salivary analysis

A 16-ml sample of saliva (without stimulation and not containing blood) was collected from each patient in a sterile container.

The salivary samples were collected at the same hour of the day (09:00 a.m. in the morning); moreover, for each patient, two salivary samples were collected at a distance of 1 week, and the average values were calculated between the two samples so as to partially reduce the subjective variability.

Four 4-ml aliquots of each sample were transferred with a plastic sterile syringe into separate centrifuge glass test tubes. The saliva sample aliquots were centrifuged in the laboratory for clinical analysis and the levels of IgA, IgG, and IgM were measured using the colorimetric immunoenzymatic method (IBL International GMBH, Germany) [10].

### Periodontal analysis

The patients were examined in a standardized manner by the same examiner who was blinded to the group assignments of the patients. For the clinical evaluation, we used dental mirrors and a University of North Carolina (UNC) periodontal probe (15 mm).

The clinical survey included the analysis of the following parameters:

– Plaque index (PI): presence of bacterial plaque on four surfaces of the tooth (distal, mesial, buccal, and lingual) in correspondence with the cervical line. A score from 0 (no plaque) to 3 (severe plaque accumulation) was assigned to each surface, and the means and standard deviations of all values were calculated (Table 1).

– Gingival index (GI): measured on four surfaces of the tooth (distal, mesial, buccal, and lingual) using a UNC periodontal probe in the gingival area adjacent to the teeth. A GI score from 0 (no gingival inflammation) to 3 (severe inflammation) was assigned, and the means and standard deviations of all values were calculated (Table 1).

– Probing depth (PD): measured from the bottom of the periodontal pocket to the gingival line using a periodontal probe inserted parallel to the long axis of each tooth, with a controlled pressure at mesial, distal, buccal, and lingual sites. For each tooth, we considered the greatest PD value measured among the four that were taken and for each subject. We calculated the percentage of sites with a value ≥ 4 mm.

– Clinical attachment level (CAL): the distance between the cemento-enamel junction and the bottom of the gingival sulcus, measured with a UNC periodontal probe inserted parallel to the long axis of each tooth, with controlled pressure at four sites (mesial, distal, buccal, and lingual). For each tooth, we considered the largest CAL value measured among the four that were taken and, for each subject, the percent of sites with a value > 3 mm were calculated, as the limit of 3 mm indicates unambiguously the presence or absence of a periodontal pocket with clinical periodontal attachment loss.

**Table 1:** Periodontal evaluation in the test group (smokers) and in the control group (non-smokers)

**Periodontal parameter**	**Test group**	**Control group**	**P value**
PI (mean ± SD)	2.2 ± 0.3	0.8 ± 0.4	< 0.05
GI (mean ± SD)	2.4 ±0.5	0.7 ± 0.3	< 0.05
PD (%)	49.3 ± 9.2	10.6 ± 2.4	< 0.05
CAL (%)	29.6 ± 4.6	3.1 ± 0.8	< 0.05

PI: plaque index.

GI: gingival index.

PD: probing depth.

CAL: clinical attachment level.

### Statistical analysis

The statistical analysis was performed using the SPSS 15.0 program. The periodontal clinical parameters were measured twice for each subject, with an inter-measurement interval of 1 week, to calculate intra-examiner reliability, and the mean value between the two measurements was considered. For each measurement, we accepted Cronbach’s alpha (α) ≥ 0.8 as indicative of good reliability.

For salivary Ig levels, plaque accumulation, and GI, we assessed statistically significant differences between the groups using Student’s *t*-test. We used the Chi-square test to compare percent values for probing depth and clinical attachment level data between the groups. The level of significance was set at *p* < 0.05. The Pearson correlation coefficient was calculated to highlight the interlinkages between the periodontal and salivary parameter values.

## Results

### Evaluation of salivary fluid

The salivary analysis revealed significantly lower Ig levels in smoking patients compared to levels in the non-smoking control group (Table 2). Specifically, IgA levels were 20.0 ± 1.2 mg/dl in smoking patients and 234.1 ± 65.2 mg/dl in controls (*p* < 0.05). IgM levels were 19.5 ± 1.6 mg/dl in the test group versus 121.0 ± 31.7 mg/dl in the control group (*p* < 0.05). Finally, IgG levels in smoking patients were 8.1 ± 1.4 mg/dl, more than two degrees of magnitude lower than levels measured in the control group (1049.4 ± 102 mg/dl; *p* < 0.05).

**Table 2:** Evaluation of salivary immunoglobulins in the test group (smokers) and in the control group (non-smokers)

**Immunoglobulins**	**Test group**	**Control group**	**P value**
IgA (mg/dl)	20.0 ± 1.2	234.1 ± 65.2	< 0.05
IgM (mg/dl)	19.5 ± 1.6	121.0 ± 31.7	< 0.05
IgG (mg/dl)	8.1 ± 1.4	1049.4 ± 102	< 0.05

### Periodontal evaluation

Analysis of the plaque accumulation findings revealed that PI scores in the test group (2.2 ± 0.3) were higher than those observed in the control group (0.8 ± 0.4; *p* < 0.05) (Table 1). With respect to gingival inflammation, smoking patients had higher GI scores (2.4 ± 0.5) than non-smoking subjects (0.7 ± 0.3; *p* < 0.05).

The percentage of periodontal sites with PD pockets ≥ 4 mm was higher (*p* < 0.05) in smoking patients (49.3 ± 9.2%) than in the non-smoking control group (10.6 ± 2.4%). Finally, our analysis of CAL revealed that smoking patients had a higher percentage of sites > 3 mm high (29.6 ± 4.6%), by almost ten-fold, than patients of the control group (3.1 ± 0.8%; *p* < 0.05).

A statistically significant correlation was observed in the test group, between each value of immunoglobulins (IgA, IgM, IgG) and the examined periodontal parameters (PI, GI, PD, CAL).

## Discussion

At present, there is strong evidence that connects several pathologies of the oral cavity with cigarette smoking, whose possible effects on the mouth are manifold, including staining of natural teeth and restorations, black hairy tongue, nicotinic stomatitis, and serious lesions such as oral carcinoma [8,11]. In particular, the association between cigarette smoking and alcohol causes an increase in the risk of occurrence of oral precancerous lesions and cancer lesions, as alcohol is capable of dissolving some carcinogenic compounds present in tobacco smoke, such as nitrosamines, and produces vasodilation, which facilitates the absorption, by the mucosa, of the toxic substances contained in the smoke [12].

Additionally, smoking can cause important alterations in saliva because saliva is the first body fluid that comes into contact with the gaseous phase of cigarette smoke. Tobacco use has been shown to cause an immediate stimulation of the salivary fluid. Although there are no documented long-term effects of this stimulation *per se*, reduced enzyme levels and buffering capacity of the saliva relative to non-smoking patients has been reported for smokers [4,13].

In this study, we detected decreased levels of IgA, IgG, and IgM in the saliva of smoking subjects; these Igs carry out a protective function against infections [2]. Additionally, despite having similar nutritional and oral hygiene habits, our smoking patients were in worse periodontal condition than control patients, demonstrating high levels of bacterial plaque, gingival inflammation, and deep periodontal pockets [14].

The high percentage of PD (49.3%), together with the presence of a lower percentage of CAL (29.6%) observed in patients of the test group, is probably due, in part, not only to the apical migration of the periodontal attachment but also to migration, in some pseudopockets, of the gingival margin in the coronal direction.

Cigarette components generate a thicker layer of bacterial plaque, which appears to be less easily removed by patients, and maintain a chronic level of inflammation of gingival tissues [15,16].

We also recorded a loss of periodontal clinical attachment in our smoking subjects that represents an irreversible process which, if not adequately treated, can lead to dental mobility and, ultimately, tooth loss due to a lack of adequate periodontal support [17]. The gingival inflammation process and periodontal destruction observed in smokers can be connected not only to the presence of bacterial plaque and of periodontal pathogenic species but also to decreases in the host’s defenses vis-à-vis a decrease in saliva Igs [18-20]. In fact, cigarette smoking can alter T-cell immunoregulation and B-cell differentiation, generating a decrease in production of Igs, which protect the oral mucosa against periodontal pathogenic bacteria. A low level of salivary Igs can be regarded as a risk factor for oral diseases, especially periodontal diseases [21,22].

Wang et al. highlighted the interconnection between periodontal disease and levels of antibodies, and in particular, that the periodontal therapy is able to determine a reduction of antibody titers of the serum against periodontal bacteria, and an increase of the antibody avidity [23].

Thus, in the presence of periodontitis, the body reacts by producing antibodies against microorganisms that are responsible for the infection; however, in the case of smoking subjects, there is a reduced number of T-helper lymphocytes that are essential for the functioning of B lymphocytes and for the production of antibodies, for which the final result is a decrease in the levels of antibodies.

Decreased IgA levels in saliva are associated with an increased degree of gingival inflammation [24]. The results of our study are aligned with those obtained by Shilpashree, who highlighted a decrease in IgA in cigarette smokers [1]. Cigarette smoking is considered to be a risk factor for the development of periodontal diseases. Among the possible pathogenetic hypotheses, the following have been considered: reduced function of gingival fibroblasts, alteration of neutrophils, and alteration of the serum antibody response against pathogenic microorganisms [25].

The reduction of immunoglobulin levels could, in some way, bind to the processes of development of periodontal lesions, although there are currently limited evidence that the salivary Igs have a protective action against periodontitis.

Furthermore, among the main limits of the study, there is the small number of examined subjects.

The results and the considerations are to be considered as preliminary findings and further studies should be conducted on a larger number of subjects in order to confirm the details of this pilot study.

The smokers, being at risk of occurrence of various oral diseases, should be monitored smoothly by a dentist.

In particular, a control of the state of periodontal health by a periodontist at regular time intervals should be performed, in order to keep under control the possible sites of periodontal infection, and implement, in the case, the required therapies.

Appropriate protocols of oral hygiene, at home and professionally, appear therefore fundamental in maintaining the health of the periodontium, even in patients who smoke [25,26].

## Conclusion

– In the smoking patients examined in this study, we detected greater bacterial plaque levels, gingival inflammation, probing depth, and loss of periodontal clinical attachment than what was found in non-smoking subjects.

– Smoking subjects also had lower levels of IgA, IgG, and IgM in their saliva than non-smoking subjects.

– The deteriorated periodontal health conditions of smoking patients can be attributed, in part, to a lowering of the host’s defense due to a decrease in the quantity of Igs in salivary fluid, despite the fact that there is little evidence that the salivary Igs have a protective action against periodontitis, and that the whole saliva examined cannot result *in toto* from the salivary glands.

## Authors’ contributions

MRG conceived the study and the sample selection; MP carried out the molecular study, followed the patients, draft the manuscript, analyzed the clinical data and conceived the discussion; ST draft the manuscript, revised the literature, analyzed the data and conceived the discussion; GG draft the manuscript; GM coordinated the research. All authors read and approved the final manuscript.
